# Comparison of efficacy of systemic antibiotics alone and combination of systemic antibiotics with gentamicin cream in diabetic foot infections

**DOI:** 10.12669/pjms.38.3.3277

**Published:** 2022

**Authors:** Munawer Latif Memon, Muhammad Ikram, Muhammad Azhar, Varda Balouch

**Affiliations:** 1 Muawer Latif Memon, Assistant Professor Surgery, POF Hospital, Wah Cantt, Pakistan; 2 Muhammad Ikram, Assistant Professor Orthopedics, POF Hospital, Wah Cantt, Pakistan; 3 Muhammad Azhar, Assistant Professor Surgery, POF Hospital, Wah Cantt, Pakistan; 4 Varda Balouch Assistant Professor Anaesthesia, POF Hospital, Wah Cantt, Pakistan

**Keywords:** Diabetic foot infection, Gentamicin, Systemic antibiotics

## Abstract

**Objective:**

To compare efficacy of systemic antibiotics alone and combination of systemic antibiotics with gentamicin cream in diabetic foot infections.

**Methods:**

Prospective Observational Study was conducted at Department of Surgery, Pakistan Ordinance Factories (POF) Hospital, Wah Cantt for duration of two years (January 2018-December 2019). A sample size of 140 diabetic foot infection patients (70 patients in each group) was calculated using WHO calculator. DFI patients were selected through non probability (consecutive) sampling technique. All patients signed consent forms before participation into study. Patients were randomly divided into two group (computer generated random number table); Group-A patients were given systemic antibiotics alone while Group-B was given combination of gentamicin cream and systemic antibiotics. SPSS version 24 was utilized for analysis purpose. Chi-square test was applied in our study. Results with p value ≤0.05 found significant.

**Results:**

Total 140 patients were included in study. There were 87(62.1%) male and 53(37.9%) females in our data. Mean age of patients was 46 years±11.3SD. Group-B (combination of gentamicin and systemic antibiotics) showed significant reduction in inflammation (p=0.03), culture negativity(p=0.001), increase clinical cure (p=0.02) and pathological eradication (p=0.03) as compared to Group-A (systemic antibiotics alone). Gender, age and duration of diabetes mellitus had insignificant association with outcomes (p>0.05).

**Conclusion:**

Diabetic foot infections are significant contributors of morbidity in our country. Combination of gentamicin cream with systemic antibiotics is highly effective in inflammation reduction, increasing clinical cure rate and pathological eradication as compared to systemic antibiotics alone in diabetic foot infections. Early identification of risk factors, proper patients care and multidisciplinary approach for diabetic foot infections prevention is required.

## INTRODUCTION

Diabetic foot infections (DFI) are most common infections associated with high morbidity, worldwide.^1^ An estimated 25 million individuals are suffering with diabetes mellitus, out of which 15% to 25% are infected with foot ulcerations.^2^ Literature reported that 50% of these ulcerations lead to increased morbidity, high rate of hospitalization and lower extremity amputation due to infections.^3^ Diabetic foot infections account for 20% of total hospital admissions in United States.^4^ In Pakistan, DFI leads to 21-48% of foot amputation due to improper management and poor glycemic controls.^5^

Pathophysiology of DFI is associated with several factors including vasculopathy, neuropathy and immunopathy. Risk factors for DFI include wounds with greater than 30 days duration, wounds with traumatic etiology, bone penetrating wounds, recurrent wounds, elevated body mass index, socioeconomic factors, duration of diabetes mellitus and presence of peripheral arterial diseases (PAD).^6^ DFI diagnosis is usually based on clinical findings of patient. Infectious disease society of America (IDSA) reported that two or more signs of inflammation (tenderness, warmth, erythema, induration and pain) and if there is no obvious purulent drainage. Diagnosis could be based upon local and systemic infections. Local signs include pain, purulent drainage, erythema, tenderness, edema and malodor; however, systemic infections include vomiting, nausea, chills, anorexia, worse glycemic control and change in mental status.^7^

There are several classifications for diabetic foot infections, however, Wagner’s classification is universally accepted grading system (0=pre ulcerative area without open lesion, 1=superficial ulcer, 2=ulcer deep to tendon, capsule, bone, 3=stage 2 with abscess, osteomyelitis or joint sepsis, 4=localized gangrene and 5=global foot gangrene). According to IDSA, treatment/management of DFI is based upon severity of infection and pathological agents.^8^

Uckay et al. reported that gentamicin sponge is effective in total clinical cure and show significant improvement in complete eradication of pathogens as compared to control (p<0.05).^9^ Uckey et al. conducted another study on gentamicin efficacy and reported that this topical antibiotic therapy achieve 91% clinical cure and 9% improvement in microbiological outcomes, however, it does not improve outcomes among patients with mild DFI.^9^ Gentamicin is an important drug in DFI treatment; however, limited data is available on its efficacy in Pakistan. Present study aims to compare efficacy of systemic antibiotics alone and combination of systemic antibiotics with gentamicin cream in diabetic foot infections.

## METHODS

This prospective observational study was conducted at Department of Surgery, Pakistan Ordinance Factories (POF) Hospital, Wah Cantt after ethical approval (Ref No.: POFHosp/Eth.com/201; dated November 13, 2020). Study duration was two (January 2018-December 2019). Sample size of 126 was rounded off to 140 DFI patients (70 patients in each group) was calculated with 95% confidence interval, power of study 84%, P1=20% and P2=15% using WHO calculator.^10^ Sampling was done with non-probability (consecutive) sampling technique. Inclusion criteria was based upon age 18-70 years, both genders and diagnosed with diabetic foot infection (based on IDSA criteria), patients with ≥1cm^2^ wound (below the malleolus), non-lactating, non-pregnant women and patients undergone any surgical intervention required for infected or necrotic tissue removal. Patients with osteomyelitis, proven ischemia on clinical examination and duplex scanning, patients with severe immune suppressions, extensive necrosis requiring amputation, peripheral arterial insufficiency requiring revascularization, infection due to any implant or foreign material insertion, patients already using gentamicin, alcohol or substance abusers were excluded from study. Diabetic foot infections were defined as presence of at least two signs of inflammation (erythema, warmth, swelling, tenderness), purulent discharge from ulcer or nearby sinus tract, along with culture positive wound swab. Clinical cure was defined as absence of at least two inflammation signs, purulent discharge and negative culture swab after intervention.^4^ Patients were randomly divided into two groups; Group-A was given systemic quinolone (ciprofloxacin 200mg 12hrly) while Group- B was given topical gentamicin cream (amount of 10g of 0.1% w/v gentamicin /gentamicin sulphate depending upon wound size) along systemic antibiotic in the form of quinolone (ciprofloxacin 200mg 12hrly). Patients were followed for seven days. Efficacy of treatment was measured in terms of inflammation reduction (50% from baseline), culture results (negative), clinical cure of infection (50% from baseline) and microbiological eradication (50% from baseline measurement) using clinical and laboratory standard institute (CLSI) guidelines. SPSS version 24 was used for data analysis. Mean and standard deviation was calculated for numerical (quantitative) data while categorical and nominal data was analyzed in terms of frequencies and percentages. Effect modifiers like age and gender were controlled using stratification process. Post stratification chi-square was utilized for measuring association between different variables. P-value ≤0.05 was reported as statistically significant difference in both interventions.

## RESULTS

Total 140 patients were included in study. There were 87(62.1%) male and 53(37.9%) females in our data. Mean age of patients was 46 years±11.3SD. There were 46(32.9%) patients in age group 18-40 years and 94(67.1%) patients in age group 41-70 years. Duration of diabetes mellitus was ≤6 months in 56(40%) and 84(60%) had >6 months of diabetes duration.

**Fig.1:**
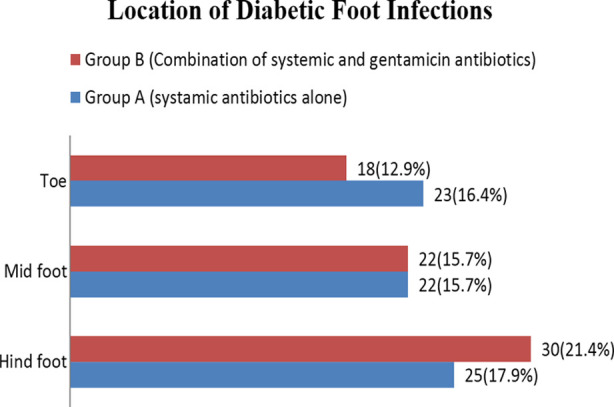
Location of diabetic foot infection.

Location of diabetic foot infection in Group-A was hind foot 17.9%, mid foot in 15.7%, and toe in 16.4% while in Group-B DFI was located in hind foot 21.4%, mid foot in 15.7% and toe in 12.9% patients as shown in Fig.1.

**Fig.2:**
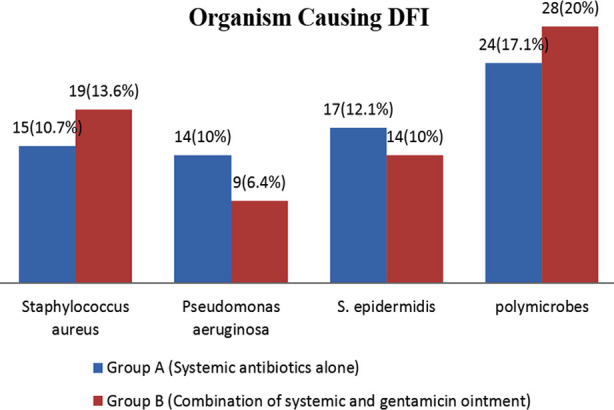
Organism causing DFI.

Organism causing DFI were staphylococcus aureus (10.7% vs 13.6%), pseudomonas aeruginosa (10% vs 6.4%), S. epidermidis (12.1% vs 10%) and polymicrobes (17.1% vs 20%) in Group-A and Group-B respectively as shown in Figure-2. Among all the patients in Group-A 70(50%), inflammation reduction was seen in 17(12.1%) and inflammation was not reduced in 53(37.9%). Among all the patients in Group-B 70(50%), inflammation was reduced in 29(20.7%) patients and not reduced in 41(29.3%) (p=0.03). In Group-A, Clinical cure was seen in 13(9.3%) and not observed in 57(40.7%) patients. In Group-B, 26(18.6%) patients show clinical cure while 44(31.4%) did not show clinical cure (p=0.02) as shown in Table-I.

**Table I:** Comparison of inflammation reduction and clinical cure in both groups.

Efficacy	Interventional Groups		Total	P-value

Inflammation reduction	Group-A (Systemic antibiotics alone)	Group-B (Combination of systemic and gentamicin antibiotic)		
No	53(37.9%)	41(29.3%)	94(67.1%)	0.03
Yes	17(12.1%)	29(20.7%)	46(32.9%)	
***Clinical cure***				
No	57(40.7%)	44(31.4%)	101(72.1%)	0.02
Yes	13(9.3%)	26(18.6%)	39(27.9%)	

Total	70(50%)	70(50%)	140(100%)	

After intervention culture results were negative in 22(15.7%) and positive in 48(34.3%) patients in Group-A while results were negative in 42(30%) patients and positive in 28(20%) patients in Group-B (p=0.001). Pathological eradication was found in 21(15%) and not eradicated in 49(35%) patients in Group-A while in Group-B pathological eradication was reported in 34(24.3%) patients and not eradicated in 36(25.7%) patients (p=0.03) as shown in Table-II.

**Table II:** Comparison of culture results and pathological eradication in both groups.

Efficacy	Interventional Groups		Total	P value

	Group-A (Systemic antibiotics alone)	Group-B (Combination of systemic and gentamicin antibiotic)		
***Culture results***				
Negative	22(15.7%)	42(30%)	64(45.7%)	0.001
Positive	48(34.3%)	28(20%)	76(54.3%)	
***Pathological eradication***				
No	49(35%)	36(25.7%)	85(60.7%)	0.03
Yes	21(15%)	34(24.3%)	55(39.3%)	

Total	70(50%)	70(50%)	140(100%)	

## DISCUSSION

Diabetic foot infections are most common complication of diabetes foot ulceration. Pakistan is among top 10 countries affected with diabetes mellitus leading to high incidence of diabetic foot infection.^11^ Jan et al. reported that foot is advise and management is an important challenge for not only diabetic patients but also for health care professionals managing diabetic foot infections.^12^

In present study, Group-B (combination of gentamicin and systemic antibiotics) showed significant reduction in inflammation as compared to Group-A (systemic antibiotics alone) (20.7% vs 12.1%, p=0.03). Chu et al reported that topical antibiotics are effective in DFI depending upon site of infection and prevent systemic side effects.^13^ Landsman et al. reported that gentamicin topical ointments are effective in small, mild and superficial diabetic foot infection, however, their efficacy is limited in severe infections.^14^

In present study, Group-B had high clinical cure rate as compared to Group-A (18.6% vs 9.3%, p=0.02). Lipsky et al. reported that gentamicin sponge is showed 35% of clinical cure. Moreover, gentamicin sponge are safe, well tolerated and without any attributed side effects.^15^ Creanor et al. reported that gentamicin had safe role in treatment of diabetic foot infection while gentamicin did not show effective results as adjunctive therapy resulting in demand of larger clinical trials.^16^

Statistically significant reduction in pathological eradication was seen in Group-B as compared to Group-A (24.3% vs 15%, p=0.03). Dumville et al. reported that total eradication of pathogens was seen in 52% patients using gentamicin collagen sponge.^17^ Varga et al. reported that gentamicin is more effective in antimicrobial eradication in topical form due to direct interaction with infected site as compared to systemic route.^18^ Another similar study reported that gentamicin ointments in combination with systemic antibiotics leads to better diabetic foot infections outcomes, however, long term efficacy of treatment is limited.^19^

In present study, Organism causing DFI were staphylococcus aureus (10.7% vs. 13.6%), pseudomonas aeruginosa (10% vs 6.4%), S. epidermidis (12.1% vs 10%) and polymicrobes (17.1% vs 20%) in Group-A and Group-B respectively. Charles et al. reported that poly microbial infections are most common in DFI (83%) including patients with Wagner grade 3 and 4.^20^ Reber et al. reported that gram negative organism *E.Coli* and *S.aureus* are most frequent pathogens associated with DFI (63% and 58% respectively).^21^ Miyan et al. reported that diabetic foot infections are associated with gram negative aerobes. They lead to conclusion that delayed referral is major cause of increasing frequency of multiple drug resistance isolates.^22^ Alavi et al. reported that in their data common causes of DFI were Escherichia Coli, Proteus vulgaris and Staphylococcus aureus with an antibiotic resistance 65%.^23^

Diabetic foot infections are leading cause of morbidity in Pakistan. There is a lot of literature available on this topic internationally. However, to the best of our knowledge this study is a unique study in Pakistan. We recommend use of systemic antibiotics in combination with gentamicin cream for diabetic foot infection treatment in resource limited areas.

### Limitation of the study

Conduction of study at single center limits generalization of study.

## CONCLUSION

Diabetic foot infections are significant contributors of morbidity in our country. Combination of gentamicin cream with systemic antibiotics is highly effective in inflammation reduction, increasing clinical cure rate and pathological eradication as compared to systemic antibiotics alone in diabetic foot infections. Early identification of risk factors, proper patients care and multidisciplinary approach for diabetic foot infections prevention is required.

### Study contribution to medical field

Diabetes is very common now in Pakistan. Diabetic foot infections lead to serious morbidity. It’s very important to understand efficacy of treatment at local level in Pakistan for diabetic foot infection. It will help the physicians to choose a treatment that is easy and feasible for diabetic patients.

### Authors’ Contribution:

**MLM:** Data collection, study designing, responsible for accuracy and integrity of study.

**MI:** Data analysis and interpretation of results.

**MA:** Data acquisition, Critical evaluation of intellectual content.

**VB:** Study write ups, Interpretation of data and critical review.
